# Long-Term Outcomes of Pouch Resizing with Ring Augmentation for Dumping Syndrome and Weight Trajectory after Roux-en-Y Gastric Bypass: A Single-Center Experience

**DOI:** 10.1007/s11695-026-08549-w

**Published:** 2026-03-03

**Authors:** Stephan Herrmann, Henrietta Pross, Gabriel Seifert, Claudia Lässle, Ning Wei, Stefan Fichtner-Feigl, Goran Marjanovic, Dalibor Bockelmann

**Affiliations:** 1 Department of General and Visceral Surgery, University Medical Center Freiburg, Freiburg, Germany; 2 Medical Strategy and Cooperations Unit, University Medical Center Freiburg, Freiburg, Germany

**Keywords:** Obesity, Metabolic surgery, Bariatric surgery, Metabolic bariatric surgery, Recurrent weight gain, Dumping syndrome, Post-bariatric hypoglycemia, Revisional surgery, Pouch resizing, Banded procedure, Ring augmentation

## Abstract

**Introduction:**

Dumping syndrome (DS) and recurrent weight gain are among the most relevant long-term challenges following Roux-en-Y gastric bypass (RYGB). Several treatment strategies have been proposed, including pouch resizing with ring augmentation (PRRA). This study presents long-term outcomes of PRRA, with a focus on weight trajectory and the management of DS symptoms.

**Methods:**

A retrospective analysis of all PRRA procedures performed at a tertiary referral center between January 2008 and September 2023. Clinical data were obtained from electronic health records and patient questionnaires.

**Results:**

Sixty-two patients (90.3 % female, mean age at time of RYGB 40.2 ± 9.5 years, mean BMI 47.4 ± 7.9 kg/m^2^) underwent PRRA at a mean interval of 56.3 ± 34.6 months after RYGB. DS was the leading indication (74.2 %), followed by recurrent weight gain (24.2 %) and primary suboptimal clinical response (1.6%). Following PRRA, 62.8 % of patients (n = 45) reported long-term improvement in DS-related symptoms, and 79 % (n = 57) achieved renewed weight loss. Long-term weight stabilization was observed, with only modest recurrent weight gain (+2.1± 5.4 kg/m^2^from post-PRRA nadir weight) after a mean follow-up of 48.6± 40.0 months. Ring removal was required in 25.8 % of patients, predominantly due to dysphagia and regurgitation.

**Conclusions:**

PRRA is an effective revisional option for managing DS symptoms after RYGB and contributes to sustained weight stability in patients with recurrent weight gain. However, ring-related adverse effects may necessitate removal in a subset of patients.

**Supplementary Information:**

The online version contains supplementary material available at 10.1007/s11695-026-08549-w.

## Key Points

PRRA reduces dumping syndrome severity and frequency long term.

PRRA supports renewed weight loss and long-term stability after RYGB weight regain.

Ring retention is high; despite adverse effects overall tolerability is favorable.

PRRA is a reversible revisional option with sustained clinical effectiveness.

**Supplementary Information:**

The online version contains supplementary material available at 10.1007/s11695-026-08549-w.

## Introduction

Metabolic bariatric surgery (MBS) is the most effective treatment option for achieving sustained weight loss in patients with obesity, while also improving obesity associated medical problems [1, 2]. Among the various surgical options, Roux-en-Y gastric bypass (RYGB) is one of the most frequently performed procedures worldwide [1, 2]. However, up to 10% of patients show a suboptimal clinical response to surgery and long-term outcomes reveal that more than one-third of all MBS patients experience significant long-term recurrent weight gain [3, 4].

To address these limitations, banded RYGB – with the placement of a non-adjustable silicone ring around the gastric pouch – has demonstrated greater long-term weight stability compared to standard RYGB [5, 6]. In patients with recurrent weight gain after RYGB, pouch resizing can lead to renewed weight loss and improvement of associated medical problems [7, 8]. Combining pouch resizing with ring augmentation (PRRA) has been proposed as an effective revisional strategy, but published data remain scarce and mostly limited to short-term follow-up [9, 10].

Beyond weight-related outcomes, MBS can also be associatied with side effects that significantly impar quality of life (QoL). Among the most impactful are dumping syndrom and post-bariatric hypoglycemia (collectively referred to as DS) [11, 12]. In severe cases, PRRA has been reported to alleviate DS symptoms, but robust evidence remains limited [13, 14].

Long-term data on the efficacy and safety of PRRA following RYGB are still lacking. The present study, reports a 15-year single-center experience with PRRA, focusing on its impact on (1) DS, (2) body weight trajectory and (3) QoL.

## Methods

### Patient population

This retrospective single-center cohort study was conducted at a tertiary referral center following formal approval by the local ethics committee. All patients who underwent revisional surgery involving pouch resizing with simultaneous placement of a non-absorbable silicone ring after prior RYGB between January 2008 and June 2023 were included. The study cohort included 16 patients who had been part of a previously conducted prospective study on the same topic [14]. Due to the retrospective nature of this study, the requirement for informed consent was waived by the institutional review board. Inclusion criteria were a history of RYGB procedure and age ≥ 18 years of age at the time of revisional surgery. Routine hospital records – including surgical charts, operative reports, discharge summaries, and outpatient clinic letters – were screened to collect relevant data. Variables extracted included sociodemographic data, dates and types of bariatric procedures, indication for revision, frequency and severity of related complaints, weight trajectory, comorbidities, and patient satisfaction. Additionally, all eligible patients were contacted and invited to complete a standardized questionnaire assessing the severity of DS, weight changes, comorbidity status, and quality of life based on the BAROS score [15]. Patients who completed the questionnaire provided written informed consent.

Diagnosis of DS was based on either clinical criteria (Sigstad score > 7 points or Arlt dumping score > 10 points) or patient-reported symptoms consistent with DS when formal scoring was not available. Due to the retrospective study design and inconsistent use of validated scoring systems, treatment response was assessed using patient-reported changes in symptom frequency and severity. In patients with DS first line treatment consisted of nutritional counseling. Further treatment options (pharmacological, endoscopic, surgical) were discussed with the patient and determined through a shared decision-making process.

Weight loss outcomes were assessed by changes in BMI, percentage of excess weight loss (%EWL), and percentage of total weight loss (%TWL). Secondary outcome measures included patient satisfaction, assessed using the BAROS score for those who completed the questionnaire. Additional outcomes included procedure-related adverse events – such as dysphagia and vomiting – as well as the rate and indication for silicone ring removal. Follow-up was defined as last patient contact following PRRA.

### Surgical Technique

Preoperative workup included endoscopy and a contrast swallow study. All revisional procedures were performed laparoscopically by one of three experienced surgeons with expertise in revisional MBS. A standardized 5-trocar technique was used, with the patient positioned in a modified lithotomy position. Adhesiolysis was routinely carried out from the candy cane limb along the gastric pouch and up to the esophageal hiatus. The gastric pouch was then resized along a 35 Fr bougie using a linear stapling device. A non-absorbable silicone ring (MiniMIZER RING, Bariatric Solutions – Stein am Rhein, Switzerland) was placed approximately 2 cm proximal to the gastrojejunostomy. A standard ring circumference of 7.5 cm was used, with intraoperative adjustment at the surgeon’s discretion (range: 6.5–8.0 cm) based on factors such as surrounding fat mass and preliminary ring fit. The ring was loosely secured around the bougie and fixed to the gastric pouch with an absorbable suture. Internal hernia orifices were inspected in all cases and closed with a two-layer non-absorbable suture, if necessary. Additional anatomical variants – such as a greatly enlarged candy cane or blind loop – were addressed at the surgeon’s discretion.

### Statistics

Continuous variables are presented as mean and standard deviation. Categorical variables are presented as frequency and percentage. All statistical analyses were performed using Stata version 18.5 (StataCorp LLC, College Station, Texas, USA).

## Results

### Patient Characteristics

A total of 62 patients were included in this retrospective study, comprising 56 females (90.3%) and 6 males (9.7%) (Table 1). The mean age at the time of RYGB was 40.2 ± 9.5 years, and the mean BMI was 47.4 ± 7.9 kg/m^2^. Prior to the index RYGB 12 patients had undergone different MBS procedures, mainly sleeve gastrectomy (66.7%) (Supplementary Table 1). According to the Edmonton Obesity Staging System (EOSS), 62.9% of patients (*n* = 39) were classified as stage 2, followed by 26.6% (*n* = 16) as stage 1, 9.7% (*n* = 6) as stage 3, and 1.6% (*n* = 1) as stage 4 (see also Supplementary Table 2). Overall, 16.1% of patients had a history of GLP1-analogue use prior to PRRA (see also Supplementary Table 3). The mean interval between RYGB and PRRA was 56.3 ± 34.6 months. The most common indication for PRRA was DS 74.2% of patients, followed by recurrent weight gain (24.2%) and suboptimal clinical response (1.6%) to RYGB (see also Supplementary Tables 4 & 5). The average length of stay following PRRA was 3.2 ± 1.3 days. The 30-day major complication rate (Clavien-Dindo grade > II) was 4.8%, with two patients (3.2%) requiring surgical intervention.

**Table 1:** Patient characteristics of patients undergoing pouch resizing with ring augmentation after Roux-en-Y gastric bypass. Continuous variables are presented as mean ± standard deviation, categorical variables as n (%). Missing values are presented as [n (%)]. BMI = body mass index; RYGB = Roux-en-Y gastric bypass; MBS = metabolic bariatric surgery; PRRA = pouch resizing with ring augmentation; DS = dumping syndrome

Total, *n* (%)	Value
	62 (100.0)
Age at time of RYGB, years	40.2 ± 9.5
Sex, n (%) - Female - Male	56 (90.3) 6 (9.7)
BMI at time of RYGB, kg/m^2^	47.4 ± 7.9
Edmonton Obesity Staging System, n (%) – Stage 1 – Stage 2 – Stage 3 – Stage 4	1–16 (26.2) 2–39 (62.9) 3–6 (9.7) 4–1 (1.6)
MBS procedures before RYGB, n (%) – 0 – 1 – 2	51 (82.3) 9 (14.5) 2 (3.2)
Alimentary limb length in cm, n (%) 100 150 180 200	3 (4.8) 42 (67.7) 1 (1.6) 4 (6.5) [13 (21.0)]
Biliopancreatic limb length in cm, n (%) 50 60 75 100 150	32 (51.2) 2 (3.2) 3 (4.8) 10 (16.1) 2 (3.2) [13 (21.0)]
Initial gastrojejunostomy size & type, n (%) 30 mm, linear stapled Unknown	45 (72.6) 17 (27.4)
GLP1-analogue use prior to PRRA	10 (16.1)
Pouch resizing with ring augmentation	
Age at PRRA Surgery, years	45.0 ± 9.9
Time interval from RYGB to PRRA, months	56.3 ± 34.6
BMI at time of PRRA, kg/m^2^	35.1 ± 6.7
Indication for PRRA, n (%) – Dumping syndrome – Recurrent weight gain – Suboptimal clinical response	46 (74.2) 15 (24.2) 1 (1.6)
Ring specific information – FOBI ring – Switched to MiniMIZER ring – MiniMIZER ring (final cohort)	2 (3.2) 2 (100.0) MiniMIZER Ring – 62 (100.0)
MiniMIZER ring size circumference cm, n (%) – 6.5 – 7.0 – 7.5 – 8.0	3 (4.8) 12 (19.4) 46 (74.2) 1 (1.6)
Length of Stay, days	3.2 ± 1.3
30-day Complications (Clavien-Dindo), n (%) – Grade II – Grade IIIa – Grade IIIb	1 (1.6) 1 (1.6) 2 (3.2)

### Effect on Body Weight

**Fig. 1:**
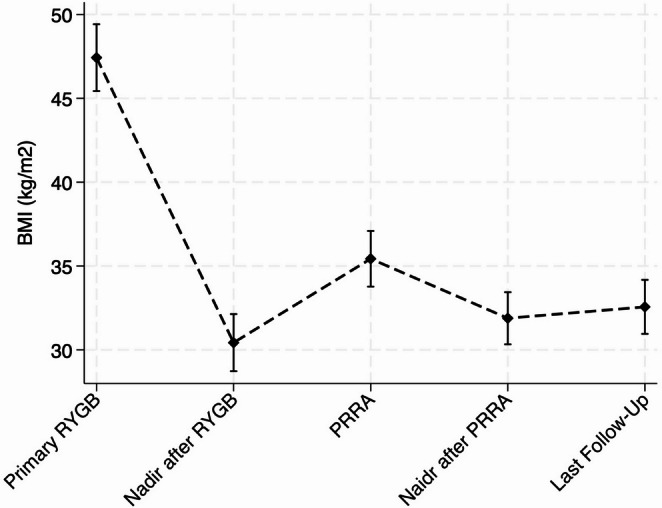
Time dependent BMI trajectory in relation to primary RYGB as well as PRRA. BMI = body mass index (kg/m2), RYGB = Roux-en Y gastric bypass, PRRA = Pouch resizing with ring augmentation

At post-RYGB nadir weight, patients had achieved an average BMI reduction of −17.2 ± 7.2 kg/m^2^, corresponding to a mean %EWL of 79.5 ± 25.1% and a %TWL of 35.5 ± 12.0% (Table 2). At time of PRRA, patients had experienced a mean BMI increase of + 5.0 ± 4.9 kg/m^2^ from their post-RYGB nadir, indicating significant recurrent weight gain – defined as a BMI increase of ≥ 5 kg/m – in 48.4% of all cases (Supplementary Table 5). Following PRRA, nearly all patients demonstrated renewed weight loss. Long-term follow-up showed weight stabilization at an average + 2.1 ± 5.4 kg/m^2^ above post-RYGB nadir BMI. Despite this, 22.6% of patients met the criteria for a long-term suboptimal clinical response – defined as %TWL < 20% (Supplementary Tables 6 and Fig. 1).

**Table 2:** Body mass index, excess weight loss and total weight loss in relation to the primary RYGB as well as PRRA. Values are presented as mean ± standard deviation. Missing values are presented as [n (%)]. BMI = body mass index (kg/m^2^); %EWL = percent excess weight loss; %TWL = percent total weight loss; RYGB = Roux-en-Y gastric bypass; PRRA = pouch resizing with ring augmentation

Time point	BMI (kg/m^2^), (*n* = 62)	%EWL (*n* = 62)	%TWL (*n* = 62)
BMI at time of RYGB	47.2 ± 7.9 [2 (3.2)]	-	-
Post-RYGB nadir BMI	30.2 ± 6.6 [1 (1.6)]	79.5 ± 25.1 [1 (1.6)]	35.5 + 12.0 [1 (1.6)]
BMI at time of PRRA	35.3 ± 6.6 [1 (1.6)]	54.2 ± 26.4 [1 (1.6)]	25.1 ± 12.5 [1 (1.6)]
Post-PRRA nadir BMI	31.8 ± 6.1 [5 (8.1)]	72.0 ± 25.2 [5 (8.1)]	32.1 ± 11.8 [5 (8.1)]
BMI at time of last follow-up	32.6 ± 6.3 [5 (8.1)]	66.9 ± 26.5 [5 (8.1)]	31.1 ± 12.8 [5 (8.1)]

### Effect on Dumping Syndrome

Following PRRA, 58.5% of all patients and 62.8% of those in the DS subgroup reported an improvement in the severity and/or frequency of DS (Table 3). Nevertheless, 32.4% and 41.2% of patients in the total cohort continued to experience DS-related symptoms on a daily or weekly basis, compared to 34.6% and 38.5% in the DS subgroup. Despite persistent symptoms, 8.8% of all patients and 7.7% in the DS subgroup reported feeling not at all restricted in daily life due to DS-related symptoms, while 26.5% and 26.9%, respectively, reported being slightly restricted, and 32.4% and 26.9% reported being only moderately restricted (see also Supplementary Table 7). Data completeness for patient-reported DS symptom frequency and perceived impact on daily life varied across outcomes.

**Table 3:** Patient-reported dumping syndrome-related symptom frequency, severity and improvement after PRRA. Information is based on patient-reported questionnaire and medical documentation. Continuous varables are presented as mean ± standard deviation or n (%). Missing values are presented as [n (%)]. DS = dumping syndrome; PRRA = pouch resizing with ring augmentation

Improvement of DS symptom severity or frequency following PRRA	Response category	Total (*n* = 62)	DS subgroup (*n* = 46)
	Worsened No change Improvement	2 (3.8) 20 (37.7) 31 (58.5) [9 (14.5)]	2 (4.7) 14 (32.6) 27 (62.8) [3 (6.5)]
Frequency of DS symptoms	Never Monthly Weekly Daily	4 (11.8) 5 (14.7) 14 (41.2) 11 (32.4) [28 (45.2)]	3 (11.5) 4 (15.4) 10 (38.5) 9 (34.6) [20 (43.5)]
Impact of DS symptoms on daily lidw	Not at all Slightly Moderate Strong Very strong	3 (8.8) 9 (26.5) 11 (32.4) 9 (26.5) 2 (5.8) [28 (45.2)]	2 (7.7) 7 (26.9) 7 (26.9) 8 (30.8) 2 (7.7) [20 (43.5)]

### Follow-up, Ring Status, Quality of Life

The mean follow-up duration after PRRA was 48.6 ± 40.0 months (Table 4 & Supplementary Table 8). At the time of last follow-up, the non-adjustable silicone ring remained in place in 46 patients (74.2%). The most common indication for ring removal was dysphagia and/or regurgitation, with a mean interval of 59.4 ± 44.3 months between PRRA and removal. Among patients with a MiniMIZER ring in situ, 61.8% reported no dysphagia and 23.5% reported mild dysphagia. Regurgitation or vomiting occurring on a monthly basis was reported by 44.1% of patients, while 34.2% reported no such symptoms. Despite the presence of these occasional symptoms, 93.9% of patients rated their QoL as moderate to very good (Table 4 and Supplementary Table 7). Data completeness for ring-associated symptoms and QoL varied across outcomes (questionnaire data available for *n* = 45).

**Table 4:** Follow-up time, ring status and ring-associated eating impairments, quality of life questionnaire. Continuous variables are presented as mean ± standard deviation and median with interquartile range, and categorical variables are presented as n (%), missings as [n (%)]. PRRA = pouch resizing with ring augmentation

Last follow-up after PRRA, months (*n* = 59)	Patients, *n* (%)
	46.6 ± 40.0 36 (15–66) [3 (4.8)]
Ring status – In place – Removed	46 (74.2) 16 (25.8)
Indication for ring removal (*n* = 16) – Dysphagia/regurgitation – Abdominal pain – Infection	14 (87.5) 1 (6.3) 1 (6.3)
Time from PRRA until ring removal, months (*n* = 16)	59.4 ± 44.3 56 (23–81)
*Questionnaire data* (*n* = 45)	
Dysphagia severity – None – Little – Moderate – Severe – Very severe	21 (61.8) 8 (23.5) 1 (2.9) 3 (8.8) 1 (2.9) [11 (24.4)]
Frequency of regurgitation or vomiting – Never – Monthly – Weekly	11 (32.4) 15 (44.1) 8 (23.5) [11 (24.4)]
Moorehead-Ardelt Quality of Life Questionnaire – Poor – Moderate – Good – Very good	2 (6.1) 8 (24.2) 11 (33.3) 12 (36.4) [12 (26.7)]

## Discussion

This study reporting one of the largest single-center PRRA cohorts with long-term follow-up is distinctive in that most patients were treated for refractory DS.

### Management of Dumping Syndrome

Initial treatment of DS focuses on dietary modifications and viscosity-enhancing agents like pectin or guar gum [11, 12]. Pharmacologic options such as acarbose, diaxozid or somatostatin analogues may can be considered in cases unresponsive to dietary interventions, although with mixed efficacy [11, 12, 16, 17]. The use of GLP-1 analogues for the treatment of dumping syndrome and postprandial hypoglycemia is an emerging approach; however, current evidence remains limited and is largely derived from case reports [17]. In severe and refractory cases, surgical or endoscopic interventions may be necessary [11, 12].

In our cohort, nearly two-thirds of all patients reported improvement in the severity and/or frequency of DS symptoms following PRRA, with approximately 11% experiencing complete resolution. This improvement was sustained over a mean follow-up period of 46.6 ± 40.0 months, indicating a durable therapeutic benefit. These findings reinforce and expand upon early data from a recent prospective study conducted at our institution that specifically focused on dumping syndrome [14]. Nevertheless, interpretation is limited by the retrospective design and the reliance on patient-reported DS-related symptoms, which may not fully capture objective changes in symptom severity following PRRA, Notably, 16.1% of patients in our cohort had a history of GLP1-receptor agonist use prior to PRRA, suggesting perceived clinical benefit despite the currently limited level of evidence. A single center study focusing on PRRA for recurrent weight gain reported DS improvement in a subgroup of patients in 75% of patients and resolution in 58% of patients one year postoperatively [13]. However, these Swiss cohorts were smaller, had shorter follow-up, and included patients with lower baseline BMIs, potentially reflecting a less severely affected population compared to that typically encountered in Germany.

Endoscopic alternatives such as transoral outlet reduction (TORe) have also demonstrated symptom resolution rates of 57% and improvement in up to 75% of cases at 1–2 years [18]. However, these findings are limited by shorter follow-up durations and smaller cohorts. Notably, patients with suboptimal clinical response to TORe in one study were subsequently referred for PRRA, suggesting that PRRA may be an effective secondary option. In cases of severe, intractable DS, bypass reversal represents an established treatment option, albeit with distinct limitations, including persistent DS symptoms, recurrent weight gain and gastroesophageal reflux disease [19]. An alternative option in patients with clinically significant recurrent weight gain is conversion to biliopancreatic diversion with duodenal switch (BPD/DS); however, available evidence remains limited, and given the technical complexity and perioperative risk profile, less invasive treatment strategies should be considered fist [20, 21].

As symptom burden and impact on quality of life (QoL) are highly individualized—and patients often adapt through coping strategies—improvements in symptom severity and reductions in daily living restrictions can lead to substantial increases in overall satisfaction, even in the presence of persistent DS symptoms.

### Recurrent Weight Gain and Suboptimal Clinical Response to RYGB

Suboptimal clinical response and recurrent weight gain remain common challenges after RYGB, with some studies estimating that up to 50% of patients may require revisional bariatric surgery [22, 23]. Although definitions vary across studies, suboptimal response is typically defined as %TWL < 20%, whereas recurrent weight gain is commonly defined as an increase in BMI of ≥ 5 kg/m² from nadir. In our cohort, only one patient met the criteria for primary suboptimal clinical response, however, 48% of patients demonstrated recurrent weight gain at the time of PRRA. Post-PRRA, approximately half of all patients experienced renewed weight loss, and nearly one-third achieved long-term weight stability. These outcomes are consistent with previous reports suggesting modest but clinically meaningful weight loss following PRRA [9, 10, 13]. TORe as another less invasive treatment option, may demonstrate greater short-term weight loss, but has also been associated with recurrent weight gain over the subsequent two years and long-term data remains limited [18, 24]. Thus, the available evidence suggests that the long-term efficacy of PRRA may be comparable—or even superior—to that of TORe. Established, although more aggressive, surgical treatment options for suboptimal clinical response or recurrent weight gain include conversion to distal gastric bypass (DRYGB) or BPD/DS [20, 21]. Reported weight loss outcomes reach up to 76% %EWL at 3 years after conversion to BPD/DS and up to 52.2% %EWL following DRYGB. However, these procedures are technically demanding and are associated with a substantially increased risk of malnutrition and long-term complications. Moreover, they may not adequately address coexisting dumping syndrome symptoms.

Recent advances in obesity pharmacotherapy have shown promising results not only in the treatment of obesity itself, but also in managing recurrent weight gain following MBS [25]. However, the overall body of evidence remains limited, and high-quality prospective data are particularly lacking.

### Patient Satisfaction and Adverse Events

This study placed particular emphasis on patient-reported outcomes, including QoL and side effects such as dysphagia and vomiting. Despite 23.4% of patients reporting weekly regurgitation or vomiting and 8.8% describing severe dysphagia, overall satisfaction remained high. Since severe DS was the most common indication for PRRA, it is important to highlight that despite the ring-associated side effects and often persistent, though significantly alleviated, DS symptoms, 69.7% of patients reported a good to very good QoL at final follow-up, while only 6% reported poor QoL. Nevertheless, interpretation of these findings is limited by the retrospective study design and the use of the BAROS score, which may lack sensitivity for detecting procedure-related adverse symptoms following PRRA. Early ring-related complications were infrequent. Two patients (3.2%) required surgical revision within 30 days—one for a local abscess and one for mechanical bowel obstruction. Both cases were managed laparoscopically, with ring removal in one. At long-term follow-up, the ring had been removed in 25.8% of patients, most commonly due to dysphagia. These removal rates and indications are in line with findings from a multicenter study of 79 PRRA patients [9].

### Limitations

This study has several limitations. First, its retrospective design, along with the reliance on electronic health records and patient-reported data, introduces potential selection and reporting biases. Most patients in our cohort underwent PRRA primarily for severe DS, which may not reflect the broader population undergoing revisional or conversion bariatric procedures. Second, the absence of standardized, serial assessments of DS using validated instruments, such as the Sigstad or Arts dumping scores, limits our ability to quantitatively evaluate changes in DS severity over time. Reliance on patient-reported DS-related symptoms may further introduce reporting bias. Similarly, the lack of repeated, formal QoL questionnaires restricts a more detailed analysis of temporal trends in patient-reported outcomes. Moreover, the QoL questionnaire used in this study was not disease-specific and was not originally designed to assess dumping syndrome–related symptoms or dysphagia following banded procedures. Consequently, the reported QoL outcomes may not fully reflect the true clinical improvement following PRRA, and the treatment effect may therefore be overestimated. Third, sociodemographic and ethnic data were not collected, limiting the generalizability of our findings across diverse populations. Nevertheless, aside from a slightly higher mean BMI at the time of the index RYGB, our cohort appears broadly representative when compared to previously published studies on PRRA.

## Conclusion

This study represents the largest cohort to date with long-term follow-up evaluating PRRA after RYGB and suggests that PRRA may provide clinically meaningful symptom improvement in selected patients with refractory dumping syndrome, along with modest and sustained benefits in weight trajectory following recurrent weight gain. While these findings are primarily based on patient-reported DS-related symptoms and a non–disease-specific quality-of-life instrument, PRRA’s full reversibility distinguishes it from other surgical and endoscopic options and may offer a practical advantage in clinical decision-making. Prospective studies incorporating validated DS-specific and disease-specific QoL measures, as well as direct comparisons with alternative interventions such as TORe, are needed to better define its role.

## Supplementary Information

Below is the link to the electronic supplementary material.

Supplementary Material 1 (DOCX 36.8 KB) 

## Data Availability

The dataset underlying this study is available from the corresponding author upon reasonable request.

## Declarations

### Use of Generative AI or AI-assisted Technologies

During the preparation of this work the authors used ChatGPT to improve readability and language. After using this tool/service, the authors reviewed and edited the content as needed and take full responsibility for the content of the publication.
