# Supplementary material for: Long-Term Outcomes of Pouch Resizing with Ring Augmentation for Dumping Syndrome and Weight Trajectory after Roux-en-Y Gastric Bypass: A Single-Center Experience
Source: Obes Surg. 2026 Mar 3;36(4):1406–13. doi: 10.1007/s11695-026-08549-w (PMC13083486; doi:10.1007/s11695-026-08549-w)
Supplement: Supplementary file 1 — Supplementary Material 1 (DOCX 36.8 KB) [file 11695_2026_8549_MOESM1_ESM.docx]

# Supplementary Material

Supplementary Table 1: Previous metabolic and bariatric surgeries prior to index Roux-en-Y gastric bypass. Values are presented as n (%).

| Type of Surgery | Number of patients (n = 12) |
| --- | --- |
| Sleeve gastrectomy | 8 (66.7) |
| Adjustable gastric banding | 3 (25.0) |
| Vertical banded gastroplasty | 1 (8.3) |

Supplementary Table 2: Prevalence of comorbidities at time of RYGB. Values are presented as n (%).

| Comorbidity | Number of patients (n = 62) |
| --- | --- |
| Type 2 diabetes mellitus | 19 (30.7) |
| Arterial Hypertension | 35 (56.5) |
| Osteoarthritis / immobility | 34 (54.8) |
| Hypothyroidism | 19 (30.7) |
| Depression | 13 (21.0) |
| Coronary artery disease | 3 (4.8) |
| Obstructive sleep apnea | 9 (14.5) |

Supplementary Table 3: Use of GLP1-analogues prior to PRRA. Values are presented as n (%).

| Indication for PRRA | GLP1-analogue use prior to PRRA |
| --- | --- |
| Total (n = 62) | 10 (16.1) |
| Dumping syndrome (n = 42) | 9 (21.4) |
| Recurrent weight gain (n = 16) | 1 (6.3) |
| Suboptimal clinical response (n = 4) | 0 |

Supplementary Table 4: Sigstad and Arts dumping scores extracted from clinical documentation, stratified by indication for PRRA. Continuous variables are presented as mean ± standard deviation, categorical values as n (%). Missing values are shown as [n (%)]. PRRA = pouch resizing with ring augmentation; DS = dumping syndrome.

| Indication for PRRA | Sigstad Score | Arts Score | Patient-reported DS, n (%) |
| --- | --- | --- | --- |
| Total (n = 62) | 12.7 ± 7.6  [28 (45.2)] | 11.9 ± 6.4  [29 (46.8)] | 21 (33.9) |
| Dumping syndrome (n = 46) | 12.6 ± 6.3  [21 (45.7)] | 12.2 ± 6.2  [22 (47.8)] | 21 (45.7) |
| Recurrent weight gain (n = 15) | 14.3 ± 10.9  [7 (46.7)] | 11.4 ± 7.7  [7 (46.7)] | not applicable |
| Suboptimal clinical response (n = 1) | 3 | 9 | not applicable |

Supplementary Table 5: Proportion of patients with recurrent weight gain and weight loss outcomes at time of pouch resizing with ring augmentation after Roux-en Y Gastric Bypass, stratified by Indication for revision. Values are presented as n (%). PRRA = pouch resizing with ring augmentation; BMI = body mass index (kg/m^2^); %EWL = percent excess weight loss; %TWL = percent total weight loss.

| Indication for PRRA | BMI ≥5 kg/m^2^ from nadir after RYGB | EWL <50 % | TWL <20 % |
| --- | --- | --- | --- |
| Total (n = 62) | 30 (48.4) | 23 (37.1) | 21 (33.9) |
| Recurrent weight gain (n = 16) | 11 (17.7) | 8 (12.9) | 7 (11.3) |
| Dumping syndrome (n = 42) | 19 (30.7) | 14 (22.6) | 13 (21.0) |
| Suboptimal clinical response (n = 4) | 0 (0.0) | 1 (1.6) | 1 (1.6) |

Supplementary Table 6: Proportion of patients with long-term suboptimal clinical response defined as %EWL < 50% and %TWL <20%, stratified by indication for revision. Variables are presented as n (%). PRRA = pouch resizing with ring augmentation; BMI = body mass index (kg/m^2^); %EWL = percent excess weight loss, %TWL = percent total weight loss.

| Indication for PRRA | TWL <20 % | EWL <50 % |
| --- | --- | --- |
| Total (n = 57) | 14 (22.6) | 19 (30.7) |
| Recurrent weight gain (n = 14) | 2 (14.3) | 2 (14.3) |
| Dumping syndrome (n = 39) | 12 (30.8) | 17 (43.6) |
| Suboptimal clinical response (n = 4) | 0 (0.0) | 0 (0.0) |

Supplementary Table 7: Quality of life assessment following PRRA: Frequency of dumping syndrome-related symptoms and their impact on daily life. Values are presented as n (%) responding to a quality-of-life questionnaire assessing both symptom frequency and perceived impact on everyday life. DS = dumping syndrome; PRRA = pouch resizing with ring augmentation.

|  | Frequency of dumping syndrome-related symptoms (n = 34) | | | |
| --- | --- | --- | --- | --- |
| Impact of dumping syndrome-related symptoms on daily life | Never | Monthly | Weekly | Daily |
| None | 1 (2.9) | 0 | 2 (5.9) | 0 |
| Little | 3 (8.82) | 4 (11.8) | 2 (5.9) | 0 |
| Moderate | 0 | 1 (2.9) | 7 (20.6) | 3 (8.82) |
| Severy | 0 | 0 | 3 (8.82) | 6 (17.6) |
| Very severe | 0 | 0 | 0 | 2 (5.9) |

Supplementary Table 8: Follow-up duration stratified by indication for PRRA. Values are presented as mean ± standard deviation. PRRA = pouch resizing with ring augmentation.

| Indication for PRRA | Last follow-up, months |
| --- | --- |
| Dumping syndrome | 41.1 ± 37.0 |
| Recurrent weight gain | 63.0 ± 45.8 |
| Suboptimal clinical response | 46 ± 44.8 |

Supplementary Table 9: Annual volume of PRRA procedures and follow-up duration. Values are presented as n (%) and median (interquartile range). PRRA = pouch resizing with ring augmentation.

| Year of PRRA | Number of patients | Last follow-up, months |
| --- | --- | --- |
| 2008 | 1 (1.6) | 119 |
| 2012 | 3 (4.8) | 135 (113–138) |
| 2014 | 1 (1.6) | 128 |
| 2016 | 6 (9.7) | 68 (66–71) |
| 2017 | 4 (6.5) | 78.5 (9.5–142) |
| 2018 | 4 (6.5) | 70.5 (66.5–77) |
| 2019 | 10 (16.1) | 48.5 (28–50) |
| 2020 | 5 (8.1) | 16 (13–36) |
| 2021 | 11 (17.7) | 32 (24–39) |
| 2022 | 11 (17.7) | 23 (9–28) |
| 2023 | 6 (9.7) | 11 (3–18) |
